# Clinical Significance of Cartilage Biomarkers for Monitoring Structural Joint Damage in Rheumatoid Arthritis Patients Treated with Anti-TNF Therapy

**DOI:** 10.1371/journal.pone.0037447

**Published:** 2012-05-21

**Authors:** Yasuo Niki, Tsutomu Takeuchi, Masanori Nakayama, Hayato Nagasawa, Takahiko Kurasawa, Harumoto Yamada, Yoshiaki Toyama, Takeshi Miyamoto

**Affiliations:** 1 Department of Orthopaedic Surgery, School of Medicine, Keio University, Tokyo, Japan; 2 Division of Rheumatology, Department of Internal Medicine, School of Medicine, Keio University, Tokyo, Japan; 3 Division of Rheumatology and Clinical Immunology, Saitama Medical Center, Saitama, Japan; 4 Department of Orthopaedic Surgery, Fujita Health University, Nagoya, Japan; Institut Jacques Monod, France

## Abstract

**Purpose:**

With the current use of biologics in rheumatoid arthritis (RA), there is a need to monitor ongoing structural joint damage due to the dissociation of articular cartilage damage from disease activity of RA. This study longitudinally analyzed levels of serum cartilage biomarkers during 54 weeks of infliximab therapy, to evaluate the feasibility of biomarkers for monitoring structural joint damage.

**Methods:**

Subjects comprised 33 patients with early RA and 33 patients with established RA. All patients received 3 mg/kg of infliximab and methotrexate for 54 weeks. Levels of the following serum cartilage markers were measured at baseline and at weeks 14, 22, and 54: hyaluronan (HA); cartilage oligometric matrix protein (COMP); type II collagen (CII)-related neoepitope (C2C); type II procollagen carboxy-propeptide (CPII); and keratin sulfate (KS). Time courses for each biomarker were assessed, and relationships between these biomarkers and clinical or radiographic parameters generally used for RA were investigated.

**Results:**

Levels of CRP, MMP-3, DAS28-CRP, and annual progression of TSS were improved to similar degrees in both groups at week 54. HA and C2C/CPII were significantly decreased compared to baseline in the early RA group (p<0.001), whereas HA and COMP, but not C2C/CPII, were decreased in the established RA group. Strikingly, serum C2C/CPII levels were universally improved in early RA, regardless of EULAR response grade. Both ΔHA and ΔC2C/CPII from baseline to week 54 correlated significantly with not only ΔCRP, but also ΔDAS28 in early RA. Interestingly, when partial correlation coefficients were calculated by standardizing CRP levels, the significant correlation of ΔHA to ΔDAS28 disappeared, whereas correlations of ΔC2C/CPII to ΔDAS28, ΔJNS, and ΔHAQ remained significant. These results suggest a role of ΔC2C/CPII as a marker of ongoing structural joint damage with the least association with CRP, and that irreversible cartilage damage in established RA limits restoration of the C2C/CPII level, even with tight control of joint inflammation.

**Conclusion:**

The temporal course of C2C/CPII level during anti-TNF therapy indicates that CII turnover shifts toward CII synthesis in early RA, but not in established RA, potentially due to irreversible cartilage damage. ΔC2C/CPII appears to offer a useful marker reflecting ongoing structural joint damage, dissociated from inflammatory indices such as CRP and MMP-3.

## Introduction

Anti-tumor necrosis factor (TNF) therapy is considered the global standard in the treatment of rheumatoid arthritis (RA), originally with the purpose of achieving clinical remission and now extending to structural remission at the radiographic level. Mounting evidence has accumulated that anti-TNF therapy not only inhibits radiographic progression of joint space narrowing, but also promotes joint space widening, particularly in patients with early RA, in whom annual changes in total modified van der Heijde (vdH)-Sharp score (TSS) are negative [1], [2]. These observations allow clinicians to expect that TNF-blockade is capable of regenerating cartilage. However, 2-dimensional radiographic assessments based on TSS have not yet confirmed whether ongoing cartilage damage can be precisely evaluated. Ultrasonography and magnetic resonance imaging have recently been reported to allow detection of subclinical joint damage in patients showing clinical remission, suggesting a dissociation between clinical remission and structural joint deterioration [2], [3]. Alternative tools that can assess ongoing joint destruction more easily than these imaging modalities should facilitate the evaluation of anti-rheumatic therapy with the potential to target structural remission. Molecular-marker technology (i.e., biomarkers) reportedly offer greater reliability and sensitivity than 2-dimensional radiography in clinical applications [4]–[6] and may offer a potential alternative to evaluate ongoing cartilage destruction in RA.

Alteration of articular cartilage turnover under arthritic conditions finally depends on the balance between the synthesis and degradation of cartilage matrix [7], [8]. This can be monitored by measuring cartilage-derived synthesis and degradation molecules released into biological fluids, such as synovial fluid, serum and urine. These cartilage-derived biomarkers have been shown to reflect structural joint damage in RA and allow assessment of therapeutic efficacy in candidate anti-rheumatoid therapy. Existing biomarkers include cartilage oligometric matrix protein (COMP), human cartilage glycoprotein-39 (YKL-40), type II collagen (CII)-related neoepitope (C2C), carboxy-terminus of three-quarter peptide from cleavage of type I collagen and CII (C1,2C), type II procollagen carboxy-propeptide (CPII), C-telopeptide of type II collagen (CTX-II), keratin sulfate (KS-5D4), and aggrecan neoepitope (CS-846). Although controversy remains about which of the biological fluids offers the best sampling source and about diurnal and activity-related variations in each biomarker [9], a fundamental principle is that markers for cartilage degradation generally increase with the progression of joint destruction, whereas markers for cartilage synthesis increase following successful treatment with anti-TNF therapy [10]. The current use of biologics in RA makes it increasingly important to identify useful and simple blood tests that can precisely reflect responses to treatment, particularly in terms of cartilage turnover and systemic inflammation resulting from RA.

Despite the advantages of technical simplicity, the practical application of serum cartilage-derived biomarkers to date has remained limited. This is due, in part, to the fact that superiority over traditional laboratory markers has not been studied in a longitudinal fashion. The present study analyzed time courses for serum levels of cartilage markers during 54 weeks of infliximab therapy in two different cohorts of early and established RA, and compared the results with other laboratory, clinical and radiographic parameters generally used for RA. This study also estimated the feasibility of using cartilage biomarkers as a potential indicator of structural joint deterioration in RA.

## Materials and Methods

All study protocols were approved by the institutional review board at Saitama Medical Center. All participants were informed about the goals and methods of the study and written consent was obtained prior to enrolment.

### Patients

In this study, a total of 66 patients were enrolled from the Division of Rheumatology and Clinical Immunology at Saitama Medical Center, and all patients fulfilled the diagnostic criteria for RA according to American College of Rheumatology criteria [11]. Thirty-three patients with arthritis symptoms of <9 months duration were classified as having early RA, and 33 patients with disease duration >10 years were classified as showing established RA. Baseline characteristics of patients are shown in Table 1. All patients had clinically active disease, despite administration of conventional first-level disease-modifying anti-rheumatic drugs, and the mean 28-joint disease activity score (DAS28)-CRP at baseline was 5.24 for early RA and 4.8 for established RA. The rate of anti-cyclic citrullinated peptide (anti-CCP) antibody was 82% (27 patients) for the early RA group and 85% (28 patients) for the established RA group. Infliximab was administered at 3 mg/kg dose in weeks 0, 2, and 6, and then every 8 weeks. MTX was concomitantly administered at 6–10 mg/week in all patients. Patients were allowed to continue use of non-steroidal anti-inflammatory drugs and oral glucocorticoids (prednisolone-equivalents <10 mg/day) that they had been taking at study entry.

**Table 1:** Baseline characteristics of the patients with early and established RA enrolled in this study*.

	Early RA (<9 months)	Established RA (>10 yrs)
**No. of patients**	33	33
**Mean age**	46.2 (19–75)	55.6 (34–80)
**Gender (male/female)**	10/23	6/27
**Disease duration [months]**	5.5 (2–9)	285 (122–516)
**Swollen joint counts**	10.3 (3–25)	10.3 (0–23)
**Tender joint counts**	8.8 (1–24)	8.6 (0–27)
**CRP [mg/dl]**	4.3 (0.2–11.0)	3.2 (0.1–10.9)
**MMP-3 [ng/ml]**	367 (31–1378)	302 (37–1292)
**Rate of anti-CCP antibody [%]**	82	85
**DAS28-CRP**	5.24 (3.11–7.75)	4.8 (2.54–6.83)
**HAQ score**	1.68 (0.75–2.38)	2.12 (0.75–3.00)
**corticosteroid administration [%(cases)]**	9 (3)	18 (6)

* Except where indicated otherwise, values are expressed as the mean (range).

Patients were evaluated for therapeutic response at baseline and 14, 22, and 54 weeks after starting infliximab. At each evaluation, blood samples were obtained and sera were separated and stored at −80°C until needed for biomarker analysis.

### Clinical evaluation of therapeutic response

The following clinical and laboratory parameters were longitudinally examined in each patient: CRP; stromelysin 1 (MMP-3); modified Health Assessment Questionnaire (HAQ) score; and DAS28-CRP. Scores for DAS28-CRP are reportedly lower than the original DAS28 assessments using the erythrocyte sedimentation rate [12] and were defined as follows: ≥4.1, high activity; ≥2.7 to <4.1, moderate activity; ≥2.3 to <2.7, low activity; and <2.3, remission. In terms of radiographic analysis, radiographs of both hands and feet at baseline and 54 weeks were available for 26 patients in the early RA group and 23 patients in the established RA group. Two expert readers independently scored articular damage and progression in a blinded fashion according to the modified vdH-Sharp scoring method. Progression of TSS from baseline to week 54 (ΔTSS) was determined, and the proportion of patients with ΔTSS≤0 was calculated.

### Cartilage biomarker analyses

The neoepitope resulting from collagenase cleavage of CII (i.e., C2C) and the c-propeptide cleaved from procollagen type II (i.e., CPII) were used as indicators of the degradation and synthesis of CII, respectively. Serum levels of each marker were measured using enzyme-linked immunosorbent assay (ELISA) (IBEX Technologies, Montreal, Quebec, Canada). The ratio C2C/CPII was used as an indicator of CII turnover, as previously reported [13], [14]. Serum COMP levels were determined by sandwich ELISA (BioVendor Laboratory, Brno, Czech Republic), using 2 monoclonal antibodies against separate antigenic determinants of human COMP molecules. Serum HA levels were determined using an HA Assay Kit (IBA method; Seikagaku, Tokyo, Japan) utilizing HA-binding protein. KS was determined by high-performance liquid chromatography (HPLC) after digestion with keratanase II (Seikagaku), as reported previously [15], [16]. Serum samples were treated with actinase E (Kaken Pharmaceutical, Tokyo, Japan) and the negatively charged substance containing KS was fractionated by Q sepharose and digested by keratanase II. These sequential enzymatic digestions yielded KS-derived β-galactosyl-(1–4)-6-O-sulfo-N-acetylglucosamine (m-ks) and β-6-O-sulfo-garactosyl-(1–4)-6-O-sulfo-N-acetylglucosamine (d-ks), which were measured using HPLC. Total KS was calculated as the sum of m-ks and d-ks values.

### Statistical analysis

Analysis of our data revealed that most of the clinical, radiographic, and laboratory results were non-parametric. Statistical comparisons of laboratory parameters or cartilage biomarker levels at each time point with those at baseline were performed using Wilcoxon's matched-pairs signed-ranks test (two-tailed). Spearman's rank correlation coefficient was used to analyze relationships between changes in individual biomarkers and changes in laboratory or functional or radiographic parameters of RA. To remove the effects of decreased inflammation (i.e., CRP level) resulting from anti-TNF therapy on cartilage turnover, partial correlation coefficients controlling for CRP level were calculated to examine the relationship between cartilage biomarkers and measures of RA disease activity. Subgroup analysis was conducted based on European League of Associations for Rheumatology (EULAR) response criteria, such as good response, moderate response, and no response. As an indicator of CII turnover, C2C/CPII ratios in each response group were analyzed longitudinally, and changes from baseline to week 54 (i.e., C2C/CPII improvement) were compared between the three subgroups using the Kruskal-Wallis test. Statistical analyses were performed using SPSS version 17.0 software (SPSS, Chicago, IL). Values of p<0.05 were considered significant.

Sample size analysis for Wilcoxon's signed-ranks test was performed to demonstrate differences between serum level at baseline and at week 54 under the effect size given in each biomarker or laboratory index. In post-hoc analysis for early RA, 11, 60, 8, 5, 13, and 22 patients would be required to demonstrate a difference with an alpha level of 0.05 and 80% power, for C2C/CPII, HA, CRP, DAS28, MMP3, and HAQ, respectively. Similarly, for established RA, 29, 30, 20, 13, 7, and 18 patients would be required to demonstrate a difference, with an alpha level of 0.05 and 80% power, for KS, HA, COMP, CRP, DAS28, and MMP3, respectively. Sample sizes for correlation analysis were also analyzed to detect a moderate to large correlation coefficient (r>0.4) that was significantly different from the presence of no correlation (r = 0) with an alpha level of 0.05 and 80% power. In post-hoc analysis for early RA at week 54, 29 and 26 patients would be required to represent a given bivariate correlation coefficient with 80% power for ΔC2C/ΔCPII vs. ΔCRP and ΔC2C/ΔCPII vs. ΔDAS28, respectively. Similarly, 31 patients would be required to detect a given partial correlation coefficient with 80% power for ΔC2C/ΔCPII vs. ΔDAS28. In the established RA at week 54, 27 and 20 patients would be required to represent a given bivariate correlation coefficient with 80% power for ΔC2C/ΔCPII vs. ΔJNS and ΔC2C/ΔCPII vs. ΔHAQ, respectively. Regarding the partial correlation coefficient, 31 patients each would be required both for ΔC2C/ΔCPII vs. ΔJNS and for ΔC2C/ΔCPII vs. ΔHAQ. Taken together with these data, the projected sample size offering sufficient statistical power was 30 patients each in the early and established RA groups.

## Results

### Clinical evaluation

Of the 33 patients in the early RA group, 1 patient achieved clinical remission and 1 patient exhibited secondary loss of efficacy after 6-month infliximab therapy. These 2 patients discontinued infliximab, and the latter patient switched to tocilizumab. One patient experienced anaphylactic reaction at week 38 and switched to etanercept. Overall, 3 patients withdrew from the study, and the remaining 30 patients in the early RA group completed 54 weeks of infliximab therapy. In the established RA group, 5 patients exhibited secondary loss of efficacy and switched to etanercept (n = 3) or tocilizumab (n = 2). One patient discontinued infliximab at week 22, because she was planning to become pregnant. Overall, 6 patients were excluded and the remaining 27 patients in the established RA group completed 54 weeks of infliximab therapy.

As expected, laboratory indices for RA disease activity, such as CRP, MMP-3 and DAS28-CRP, had decreased significantly by week 54 in both groups (Table 2). The decrease in DAS28-CRP was prominent in patients with early RA, with mean score at week 54 below the level of clinical remission. Mean HAQ score was significantly decreased at week 54 in the early RA group, but remained unchanged in the established RA group. When DAS28-CRP scores were assessed using EULAR response criteria, 90% and 78% of patients were categorized as showing good or moderate response in the early and established RA groups, respectively, with no significant difference apparent between groups. Radiographic structural assessment using the TSS revealed that mean ΔTSS per year (annual progression) was 3.7 in the early RA group and 4.0 in the established RA group, while the proportion with ΔTSS≤0 exceeded 70% in both groups, suggesting that our clinical study using infliximab yielded successful clinical results comparable to those in a previous study in Japan [17].

**Table 2:** Time-course changes in biochemical, clinical, radiographic, and functional measures during 1-year infliximab therapy.

	Time after starting infliximab			
	0W (baseline)	14W	22W	54W
**Early RA (n = 30)**				
CRP [mg/dl]	4.12†	1.43**	1.02**	0.45**
DAS28-CRP	5.16	3.13**	2.74**	2.2**
MMP-3 [ng/ml]	342	167	116*	105*
HAQ score†	1.46	0.92**	0.9**	0.8**
TSS (SD) (n = 26)	10.5 (18.7)	n.d.***	n.d.	14.2 (20.1)
JNS (SD) (n = 26)	4.8 (7.6)	n.d.	n.d.	7.2 (10.3)
ΔTSS (mean/median)				3.7/0
Rate of ΔTSS≤0 [%(cases)]				73 (19)
EULAR category of response [% (cases)]				
Good				63 (19)
Moderate				27 (8)
No response				10 (3)
**Established RA (n = 27)**				
CRP [mg/dl]	2.91	0.68**	0.66*	0.66*
DAS28-CRP	5.11	2.96**	2.76**	2.80**
MMP-3 [ng/ml]	298	92	98*	91*
HAQ score	1.88	1.7	1.71	1.73
TSS (SD) (n = 23)	211.2 (90.2)	n.d.	n.d.	215.4 (96.3)
JNS (SD) (n = 23)	85.8 (43.6)	n.d.	n.d.	88.1 (44.2)
ΔTSS (mean/median)				4.0/0
Rate of ΔTSS≤0 [%(cases)]				70 (16)
EULAR category of response [% (cases)]				
Good				41 (11)
Moderate				37 (10)
No response				22 (6)

† Except where indicated otherwise, values are expressed as the mean.

* p<0.05 versus baseline levels.

** p<0.001 versus baseline levels.

*** n.d., not determined.

### Temporal changes in cartilage biomarkers during 54-week infliximab therapy

**Figure 1:**
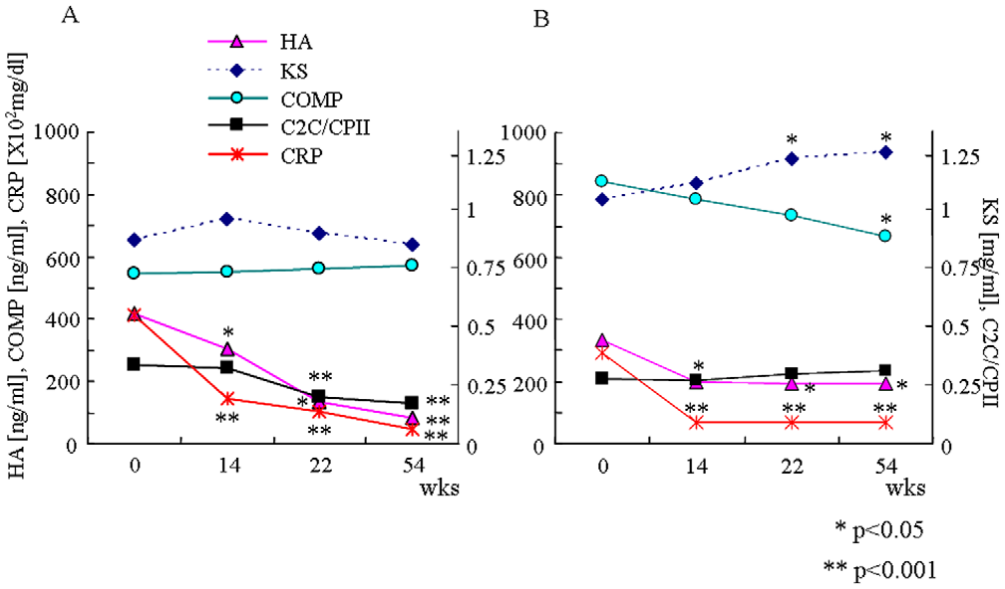
Temporal course of cartilage biomarker levels during 54-week infliximab therapy. Data for each time point represent mean levels of serum CRP, HA, COMP, KS, and C2C/CPII in early RA (A) and established RA (B). Standard deviation (SD) error bars are not plotted in these graphs for clarity and are shown in Table 3. Statistical analyses were performed using Wilcoxon's matched-pairs signed-ranks test, two-tailed. *p<0.05 versus level at baseline. **p<0.001 versus level at baseline.

In the early RA group, serum levels of HA and C2C/CPII gradually decreased over time during 54-week infliximab therapy, and levels of HA at weeks 14, 22 and 54, and C2C/CPII at weeks 22 and 54 were significantly lower than each baseline level (p<0.001). These two biomarkers appeared to synchronize with decreasing CRP level over the 54 weeks of infliximab therapy. In contrast, COMP level remained constant during infliximab therapy (Table 3, Fig. 1A). Serum KS level slightly increased at week 14, followed by a gradual decrease to the baseline level at week 54. In the established RA group, serum level of HA was significantly decreased at week 14 (p<0.05) and became constant, demonstrating a quite similar pattern to that of CRP, whereas C2C/CPII remained unchanged during 54 weeks (Table 3, Fig. 1B). Level of serum COMP in established RA, which demonstrated a higher baseline level than in early RA, gradually decreased during the 54-week infliximab therapy with significant differences at week 54 (p<0.05). In contrast, level of serum KS in established RA, which also demonstrated a higher baseline level than in early RA, gradually increased with significant differences at weeks 22 and 54 compared to baseline (p<0.05).

**Table 3:** Time-course changes in the levels of cartilage biomarkers during 1-year infliximab therapy.

	Time after starting infliximab			
	0W (baseline)	14W	22W	54W
**Early RA (n = 30)**				
HA [ng/ml]	420 (923) †	306 (852)*	134 (166)*	81 (69)**
KS [µg/ml]	0.87 (0.30)	0.96 (0.37)*	0.90 (0.31)	0.85 (0.22)
COMP [ng/ml]	545 (297)	549 (237)	561 (232)	570 (239)
C2C [ng/ml]	229 (47)	204 (45)	171 (46)*	156 (46)**
CPII [ng/ml]	733 (304)	858 (437)	875 (416)*	997 (489)*
C2C/CPII	0.34 (0.17)	0.32 (0.16)	0.20 (0.04)**	0.17 (0.05)**
**Established RA (n = 27)**				
HA [ng/ml]	335 (301)	199 (209)*	191 (196)*	193 (199)*
KS [µg/ml]	1.05 (0.34)	1.12 (0.43)	1.22 (0.38)*	1.25 (0.46)*
COMP [ng/ml]	845 (321)	788 (278)	734 (267)	669 (230)*
C2C [ng/ml]	224 (68)	231 (62)	211 (58)	264 (54)
CPII [ng/ml]	1039 (465)	1087 (439)	834 (306)	886 (243)*
C2C/CPII	0.28 (0.15)	0.27 (0.13)	0.3 (0.11)	0.31 (0.12)

† Values are expressed as mean (SD).

* p<0.05 versus baseline levels.

** p<0.001 versus baseline levels.

### Correlations between cartilage biomarkers and RA disease activity markers

Correlations between levels of cartilage biomarkers and degree of RA disease activity (e.g., CRP, MMP-3, and DAS-28), radiographic progression (e.g., ΔJNS) and patient function (e.g., HAQ score) were investigated at weeks 22 and 54. Several marker pairs with significant correlations are summarized in Table 4. Among the four cartilage biomarkers tested, only C2C/CPII and HA level yielded strong linear correlations with several disease activity measures of RA. Since the degree of structural joint damage, particularly in terms of cartilage destruction, is reportedly dissociated from the degree of joint inflammation, the present analysis focused on whether temporal changes in cartilage turnover were associated with the degree of CRP decrement. In the early RA group, ΔC2C/ΔCPII and ΔHA displayed significant correlations with ΔCRP at both weeks 22 and 54. Correlation with ΔDAS28 was observed at week 22 for ΔHA, and at both weeks 22 and 54 for ΔC2C/ΔCPII. Interestingly, according to partial correlation coefficients, the significant correlation between ΔHA and ΔDAS28 disappeared when the level of CRP was standardized. In contrast, the significant correlation between ΔC2C/ΔCPII and ΔDAS28 remained present even after standardization of CRP levels. In the established RA group, ΔC2C/ΔCPII correlated with neither ΔCRP nor ΔDAS28, whereas ΔHA did correlate with ΔCRP at both weeks 22 and 54. Of note is the finding that ΔC2C/ΔCPII significantly correlated with ΔJNS and ΔHAQ at week 54, and these significant correlations were present even after standardizing CRP level. These results suggest that ΔHA preferentially correlated with the level of CRP, while ΔC2C/ΔCPII represented a CRP-independent indicator of joint destruction reflecting radiographic joint space narrowing and patient function.

**Table 4:** Spearman's correlation coefficients and partial correlation coefficients of cartilage markers vesus RA disease markers.

		22W		54W	
		Spearman's r	partial r**	Spearman's r	partial r
**Early RA**					
**ΔC2C/ΔCPII**	vs. ΔCRP	0.44 (0.02)*	n.a.¶	0.50 (0.01)	n.a.
	vs. ΔDAS28	0.49 (0.03)	0.48 (0.04)	0.52 (0.01)	0.49 (0.02)
	vs. ΔKS	n.s.	n.s.†	−0.46 (0.03)	n.s.
**ΔHA**	vs. ΔCRP	0.37 (0.04)	n.a.	0.45 (0.03)	n.a.
	vs. ΔDAS28	0.52 (0.02)	n.s.	0.43 (0.04)	n.s.
	vs. ΔMMP-3	0.63 (0.02)	n.s.	0.74 (0.002)	0.48 (0.04)
**Established RA**					
**ΔC2C/ΔCPII**	vs. ΔCRP	n.s.	n.a.	n.s.	n.a.
	vs. ΔDAS28	n.s.	n.s.	n.s.	n.s.
	vs. ΔKS	n.s.	n.s.	−0.49(0.03)	n.s.
	vs.ΔJNS	n.d.	n.d.‡	0.51 (0.03)	0.48 (0.04)
	vs.ΔHAQ	n.d.	n.d.	0.58 (0.02)	0.49 (0.03)
**ΔHA**	vs. ΔCRP	0.56 (0.02)	n.a.	0.43 (0.04)	n.a.
	vs. ΔDAS28	n.s.	n.s.	n.s.	n.s.
	vs. ΔMMP-3	0.49 (0.04)	n.s.	0.51 (0.04)	n.s.

* Values are correlation coefficients calculated using Spearman's rank correlation. P values are expressed in parentheses. p<0.05 is considered as statistically significant.

** Partial correlation coefficients were obtained after controlling CRP level for each marker pair.

¶ n.a., not applicable.

† n.s., not significant.

‡ n.d., not determined.

### Association between balance of CII synthesis/degradation and efficacy of infliximab

**Figure 2:**
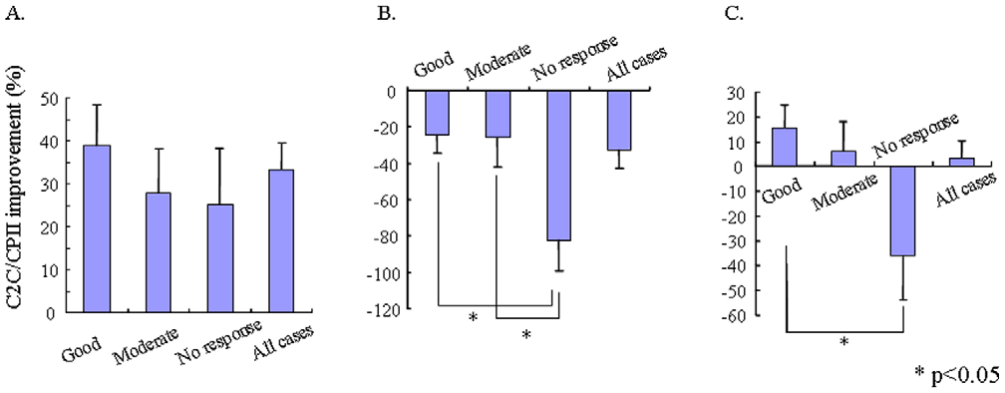
Improvement of C2C/CPII from baseline to week 54 was assessed in early RA (A), established RA (B), and all patients (C). Data are expressed as mean (± SD) percentage of baseline. Patients were divided into three subgroups according to the degree of clinical response at week 54 using EULAR response criteria. Positive values signify that the balance of CII synthesis/degradation is biased toward synthesis, while negative values indicate that the balance is biased toward degradation. Statistical analysis was performed using the Kruskal-Wallis test. *p<0.05.

As C2C/CPII preferentially reflected joint damage independent of changes in inflammatory indices, C2C/CPII was further analyzed for relationships with EULAR response grade after 54 weeks of infliximab therapy. Strikingly, in the early RA group, C2C/CPII was reduced (i.e., improved), regardless of responsiveness to infliximab, indicating that even in non-responders, the balance of CII synthesis/degradation became shifted toward synthesis (Fig. 2A). By contrast, C2C/CPII in the established RA group universally increased (i.e., worsened), regardless of responsiveness to infliximab, indicating that the net balance of CII synthesis/degradation was shifted toward degradation even in good responders (Fig. 2B). For all patients, C2C/CPII in non-responders was increased (i.e., worsened) compared to baseline, whereas C2C/CPII in moderate or good responders was reduced (i.e., improved) from baseline (Fig. 2C).

## Discussion

RA is an inflammatory joint disease that predominantly involves the synovial tissues of joints and is characterized by variable disease onset and clinical course, ultimately resulting in structural joint destruction and subsequent physical disability. Early treatment with anti-TNF therapy is currently accepted as an effective strategy to achieve clinical and structural remission, potentially improving physical disability. In the present study, 54-week treatment with infliximab achieved satisfactory results according to the levels of CRP, MMP-3, and DAS28, EULAR response criteria, and the rate of ΔTSS≤0. Although these clinical measures for RA were similarly improved in both early and established RA, C2C/CPII as an indicator of CII turnover was significantly improved from baseline in early RA, but not in established RA. Strikingly, C2C/CPII was universally improved and shifted toward CII regeneration in early RA, regardless of EULAR response grade. In contrast, C2C/CPII was universally shifted toward CII degradation in established RA, regardless of EULAR response grade. From the perspective of CII turnover, anti-TNF therapy should clearly be initiated while the patient is still in the early phase, while the regenerative capacity of articular cartilage is maintained and before irreversible structural joint damage occurs. Past clinical trials, such as the Best study, have demonstrated that patients initially treated with infliximab exhibited persistent low disease activity even after the cessation of infliximab, suggesting the clinical significance of early introduction of aggressive treatment in early RA with poor prognostic factors [18].

A noteworthy finding was that annual changes in cartilage biomarker levels correlated with annual progression of joint destruction and physical function after anti-TNF therapy. To the best of our knowledge, no previous studies have provided such insights. Significant correlations were found between ΔC2C/CPII and ΔHAQ (r = 0.58, p = 0.02) and between ΔC2C/CPII and ΔJNS (r = 0.51, p = 0.03) in our established RA cohort. The fact that joint space narrowing on radiography largely reflects loss of cartilage rather than bony erosion may explain the close relationship between ΔJNS and ΔC2C/CPII. Although determining whether HAQ improvement is a cause or consequence of decreased ΔC2C/CPII is difficult, one potential explanation for this correlation is that high activity and subsequent mechanical loading on cartilage either resulted in or is attributed to improved CII turnover, as reported by Roos et al. [19].

Serum levels of KS have been reported as an indirect measure of aggrecan turnover in articular cartilage and further analyzed for a role as a predisposing factor for osteoarthritis (OA) with a polyarticular, progressive phenotype [20]. KS level is elevated not only in patients with cartilage degeneration, but also in healthy individuals with higher sports activity [21], indicating that KS level can universally elevate in cases with increased cartilage turnover, even in normal cartilage. Wakitani et al. have reported that serum KS level in the knee OA is elevated more in the early stage than in the advanced stage, suggesting that KS reflects aggrecan turnover rather than the degree of joint destruction [16]. Similarly, levels of serum CS-846 epitope, as the marker for newly synthesized aggrecan, have been shown to increase in slowly progressive RA and signify an ability or attempt to repair damaged cartilage matrix [22], [23]. From this perspective, gradually increased KS turnover in established RA was potentially attributable to not only persistent aggrecan release from cartilage, but also the fact that newly synthesized aggrecan cannot be incorporated into cartilage matrix that has been inherently damaged at baseline. In cases of early RA, KS levels were increased in week 14, but stabilized thereafter due to the inhibitory effects of TNF blockade on cartilage degradation, leading to normalization of cartilage turnover.

Contrasting results were obtained regarding the temporal course of serum COMP levels between early and established RA. Numerous studies have proposed the feasibility of serum COMP levels in monitoring articular cartilage damage or predicting the efficacy of anti-TNF therapy in RA [24]–[26]. In our established RA cohort, serum COMP levels were high at baseline, and gradually decreased during the course of infliximab therapy, as previously reported [24]. However, in early RA, serum COMP levels at baseline were low, and remained unchanged over 54-week infliximab therapy, despite fully exertion of the therapeutic effects of infliximab. Given the evidence that serum COMP levels elevate with increasing physical activity [27], constant levels of COMP over time in early RA might theoretically be explained if the decrement in COMP levels induced by infliximab is balanced by increased physical activity as evidenced from decreased HAQ scores.

Most measures of RA disease activity, such as the simplified disease activity index, Boolean criteria, and DAS28, exhibit correlations with CRP, because CRP is involved in each definition. As for cartilage biomarkers, this study showed that ΔHA and ΔC2C/CPII correlated significantly with not only ΔCRP, but also ΔDAS28 in early RA. Interestingly, when partial correlation coefficients were calculated by standardizing CRP levels, the significant correlation of ΔHA with ΔDAS28 disappeared, whereas correlations of ΔC2C/CPII with ΔDAS28, ΔJNS, and ΔHAQ were still significant. These results suggest a role of ΔC2C/CPII as a marker of ongoing structural joint damage with the least association to markers for systemic inflammation, such as CRP and erythrocyte sedimentation rate. Indeed, serum cytokine profile among the patients with established RA in this study revealed that levels of most inflammatory cytokines, including IL-6, TNF, and IL-17, were decreasing with decreasing CRP level over 54-week of infliximab therapy, whereas C2C/CPII level deteriorated over time (unpublished data, Fig. S1).

Significant concerns remain as to the differences in cartilage regenerative capacity between early and established RA. C2C/CPII was universally improved and shifted toward CII regeneration in early RA, but not in established RA, regardless of responsiveness to infliximab. Restoration of C2C/CPII balance and the resulting cartilage regeneration is likely to be relevant to the degree to which the cartilage matrix has been damaged before starting anti-TNF therapy, rather than the magnitude of the suppression of systemic inflammation during anti-TNF therapy. A previous experimental study showed that mice with antigen- or zymosan-induced arthritis displayed reversible cartilage damage only when levels of collagen degradation were low [28]. This finding was corroborated by a human study using cartilage explants culture in vitro, in which aggrecanase-mediated aggrecan degradation did not influence the regenerative capacity of cartilage, but was markedly impaired after MMP-mediated aggrecan and collagen type II degeneration were initiated [29]. MMP-mediated aggrecan and collagen type II degeneration might thus represent a turning point for the reversibility of cartilage degradation. Therefore, whether RA is in the early or established phase (i.e., disease duration) does not appear critical.

In conclusion, ΔC2C/CPII offers a useful marker reflecting ongoing cartilage damage, which appears dissociated from inflammatory indices. As most measures of RA disease activity generally correlate with CRP, C2C/CPII appears to be of great clinical value as a CRP-independent marker, particularly when ongoing structural joint damage is evaluated during biological therapy in RA. The temporal course of C2C/CPII level during anti-TNF therapy indicated that CII turnover shifted toward CII synthesis in early RA, but not in established RA, potentially due to irreversible cartilage damage. The clinical significance of C2C/CPII should be further investigated in large-scale prospective studies to evaluate the feasibility of using this ratio as a surrogate marker for monitoring ongoing structural joint damage during the course of anti-rheumatoid therapy.

## Supporting Information

Figure S1 Temporal course of the serum levels of various cytokines in patients with established RA during 54-week infliximab therapy. The data were measured using a Luminex® multiplex beads cytokine assay. Values were expressed as a proportion of each baseline value. Of note is the finding that serum levels of most inflammatory cytokines, including IL-6, TNF, and IL-17, were decreasing with decreasing CRP level over 54-week of infliximab therapy, whereas C2C/CPII level deteriorated over time.
(TIF)
