# Supplementary figures and images for: Clinical Significance of Cartilage Biomarkers for Monitoring Structural Joint Damage in Rheumatoid Arthritis Patients Treated with Anti-TNF Therapy
Source: PLoS One. 2012 May 21;7(5):e37447. doi: 10.1371/journal.pone.0037447 (PMC3357428; doi:10.1371/journal.pone.0037447)

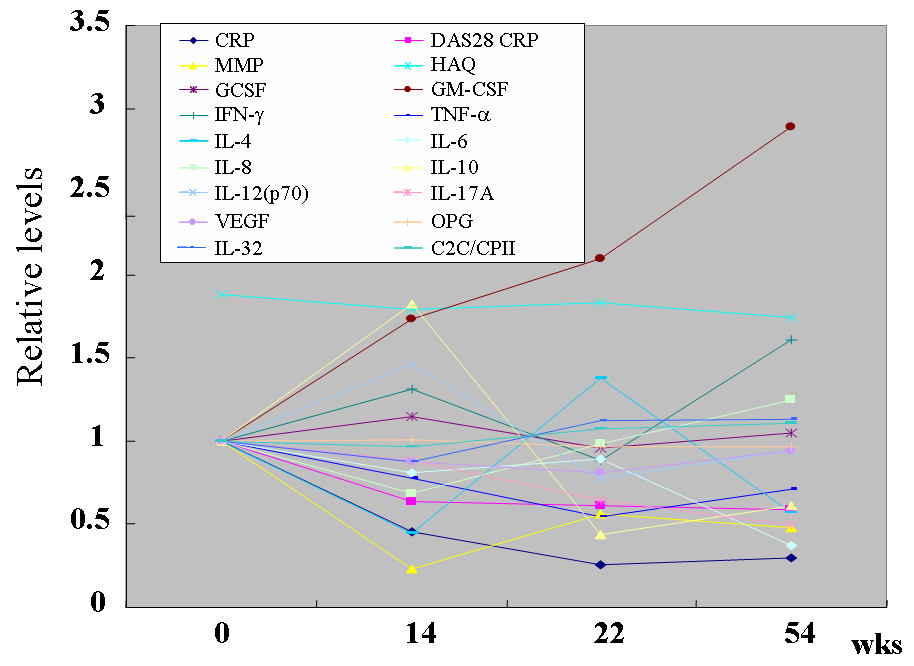

Supplement: Figure S1 — Temporal course of the serum levels of various cytokines in patients with established RA during 54-week infliximab therapy. The data were measured using a Luminex® multiplex beads cytokine assay. Values were expressed as a proportion of each baseline value. Of note is the finding that serum levels of most inflammatory cytokines, including IL-6, TNF, and IL-17, were decreasing with decreasing CRP level over 54-week of infliximab therapy, whereas C2C/CPII level deteriorated over time. (TIF) [file pone.0037447.s001.tif]
